# P006: Highlights in bloodstream infections: where does the patient acquire the infection?

**DOI:** 10.1186/2047-2994-2-S1-P6

**Published:** 2013-06-20

**Authors:** M Rodriguez-Aguirregabiria, T Gimenez-Julvez, J Rodriguez-Aguirregabiria, M Villanova, P Rico Cepeda, E Palencia Herrejon

**Affiliations:** 1 Hospital Universitario Infanta Leonor, Madrid, Spain

## Introduction

Bloodstream infections (BSI) still account for significant morbidity and mortality.

## Objectives

The objective of this study was to describe the epidemiology, etiology, sources and adequacy of empiric antimicrobial treatment in BSI.

## Methods

A retrospective study about all BSI diagnosed during one year. The pattern resistant pathogen study was EPINE-EPPS project.

## Results

340 patients were included. Median age was 74.5 years [interquartile range (IQR), 58.5-80.5]; acute physiology and chronic health evaluation (APACHE II) score was 13 (IQR, 7-29).

BSI were community-acquired in 56% of the cases. The most common source of BSI was urinary tract (48.3%), intra-abdominal (25.6%) and lower respiratory tract infections (18.6%). The most commonly isolated microorganisms were: *Escherichia coli*, *K. pneumoniae*, *S.aureus* (15% oxacilin resistant) and *S. pneumoniae*. The 8.6% of enterobacteracea family produced extended-spectrum B-lactamasas (ESBLs). Inappropriate treatment was observed in 24.5% and crude mortality rate was 7.7%.

28% BSI were nosocomial-acquired. The sources of BSI were unknown in 31.7% of the cases and catheter-related in 25.7%. The secondary sources of BSI were intra-abdominal in 57% of the cases. The most common isolated microorganisms were: *S.epidermidis* and other coagulasa negative, *Candida*, *S. aureus* (36% oxacilin resistant) and *E. coli*. 25% of enterobacteracea family were ESBLs. We found 5 BSI caused by *Acitetobacter* carbapenem (CPM) resistant and 2 BSI by *P. aeruginosa* CPM resistant. Inappropriate treatment was observed in 52.5% and mortality rate was 28.7%.

Health-care related BSI produced 15.1% of the cases. The source of BSI were unknown in 22.6% and catheter- related in 11.3%. The secondary sources of BSI were urinary tract (60%), intra-abdominal (31.4%) and respiratory tract infections(8.6%). The most common microorganisms were: *E.coli*, *S.aureus* (25% oxacilin resistant), *S.epidermidis* and *K.pneumoniae*. Inappropriate treatment was noticed in 34% and mortality rate was 17%.

## Conclusion

The knowledge of local epidemiology is a capital information to improve empiric antimicrobial treatment and to reduce mortality-related inappropriate treatment.

## Disclosure of interest

None declared

